# Missed opportunities in the way medical schools evaluate the ethical domain in clerkship rotations

**DOI:** 10.1371/journal.pone.0217717

**Published:** 2019-05-29

**Authors:** Maria Fernanda dos Santos, João F. L. Schoueri, Camila T. Vidal, Pedro T. Hamamoto Filho, Fernanda B. Fukushima, Edison I. O. Vidal

**Affiliations:** 1 Public Health Department, Botucatu Medical School, Sao Paulo State University (UNESP), Botucatu, SP, Brazil; 2 Occupational Therapy Department, University of Sorocaba (UNISO), Sorocaba, SP, Brazil; 3 Internal Medicine Department, Botucatu Medical School, Sao Paulo State University (UNESP), Botucatu, SP, Brazil; 4 Neurology, Psychology and Psychiatry Department, Botucatu Medical School, Sao Paulo State University (UNESP), Botucatu, SP, Brazil; 5 Anesthesiology Department, Botucatu Medical School, Sao Paulo State University (UNESP), Botucatu, SP, Brazil; Universidade de Mogi das Cruzes, BRAZIL

## Abstract

**Background:**

Several lines of evidence indicate that medical schools have been failing to adequately nurture empathy and the ethical dimension in their graduates, the lack of which may play a central role in the genesis of medical errors, itself a major source of avoidable deaths, incapacity and wasted resources. It has been widely proposed that medical schools should adopt evaluation strategies as a means to promote a culture of respectful relationships. However, it is not clear if evaluation strategies in medical schools have addressed key domains related to that aim, such as ethics, through the perspective of their students. Hence, we conducted a national survey of instruments used by Brazilian medical schools to assess clerkship rotations from the perspective of students, with a main focus on the ethical domain.

**Methods:**

The authors invited 121 randomly selected institutions to participate in the study. Key informants answered a questionnaire about clerkship rotations and sent copies of any instrument used to assess the quality of clerkship rotations according to the students’ perspectives.

**Results:**

Twenty-six (53%) of 49 participating schools used an instrument to assess the quality of clerkship rotations according to the perspective of students. Just 13 (27%) schools had instruments containing at least one question encompassing the ethical domain. Only 2 (4%) schools asked students specifically about the occurrence of any negative experience concerning the ethical domain during rotations. Merely 1 (2%) school asked students about having witnessed patient mistreatment and none asked about mistreatment against students themselves.

**Conclusions:**

There are several missed opportunities in the way medical schools assess the quality of clerkship rotations regarding the ethical domain. Closing the gap between usual institutional discourses regarding ethics and how that dimension is assessed within clerkship rotations might represent an important step towards the improvement of medical education and healthcare systems.

## Introduction

Several lines of evidence indicate that medical schools have been failing to adequately nurture empathy and the ethical dimension in their graduates [1–5]. In 1994 Feudtner et al [6] coined the term “ethical erosion” to denote a process that affects many medical students during their training and which is characterized by the abandonment of certain moral values, changes in attitude and in ethical identity. In alignment with that perception, Leape and colleagues [3,7] made a strong argument that the healthcare field is paved with a diffuse culture of disrespect which plays a central role in the genesis and maintenance of medical errors, itself a major source of avoidable deaths, incapacity and wasted resources [8].

Most experts identify the phenomena of declining empathy and ethical erosion during medical education as a consequence of the hidden curricula of healthcare organizations [2,9,10]. The hidden curriculum has been defined as the learning derived from the cultural and organizational environment of institutions [11,12]. It consists of unwritten, taken-for-granted rules and customs that provide lessons about what is important, valued and acceptable or not in the relationships with patients and colleagues on a daily basis. Negative examples that medical students and residents observe during their training represent the dimension of the hidden curriculum that plays the most important role in the phenomena of declining empathy and ethical erosion [2,6,9,11,13–16].

It has been proposed that to change that picture for the better, medical schools should adopt evaluation strategies that foster a cultural environment of respectful and trustworthy relationships among professionals, students and patients [7,12,17]. The rational behind this proposal is that evaluation strategies are an important driver of change in institutions as administrators and staff may value them just as students consider carefully what is expected of them to succeed in their finals [12,18–20]. Despite the apparent logic of those arguments there is a significant gap in the knowledge base concerning if and how medical schools have been using evaluation strategies with those aims. As a matter of fact, we were not able to identify any report of any survey of medical schools examining such strategies. Hence, we conducted the present survey to examine the instruments used by Brazilian medical schools to assess clerkship rotations from the perspective of students, with a main focus on items associated with the ethical domain. Our main hypothesis was that in most cases the available instruments would not address possible experiences of unethical situations and behaviors during those rotations.

## Materials and methods

We conducted a national survey of instruments used by Brazilian medical schools to evaluate clerkship rotations from the perspective of medical students. Data collection took place between August 2014 and September 2015. Every medical school that had at least one group of students graduated by the first semester of 2014 was eligible to participate in the survey. In May 2014 there were 176 medical schools in Brazil that fulfilled that criterion.

We calculated a sample size of 121 schools to attain a margin of error of 5% and a confidence interval of 95% considering a response distribution of 50% regarding the presence of instruments assessing the ethical dimension during clerkship rotations [21]. The selection of medical schools to participate in this study proceeded as follows. We extracted a list of all medial schools in Brazil from the website of the Brazilian Ministry of Education and organized those schools in alphabetical order in a numbered electronic spreadsheet. We then used a computer-based random number generator to produce a sequence of numbers corresponding to the schools to be invited to participate in the study.

We used multiple strategies to invite medical schools to participate in the study, as follows:

- We asked the Brazilian Medical Education Association to send an electronic invitation for its member schools.
- We made phone calls and sent invitation e-mails to the administration offices of each medical school.
- We asked for the assistance of members of the Foundation for the Advancement of International Medical Education and Research in Brazil through their e-mail server.
- We also contacted representatives of medical schools in person during a regional meeting of the Brazilian Medical Education Association.

Once we managed to establish communication with the administration office of a medical school, we sent them e-mail containing the consent form to be signed by a representative of that medical school, and a questionnaire to be answered by a key informant appointed by the administration office of each medical school.

We performed at least 5 communication attempts in different days and hours before classifying a school as non-respondent in the study. Once we achieved communication by phone or e-mail with the administration office of a medical school, we waited about one week to contact it again asking for an update concerning its participation in the study. If no feedback was provided, we made further weekly contact attempts with phone calls and/or e-mails for at least 5 weeks before we discarded a medical school from the study.

The questionnaire asked for information concerning the duration of clerkship in each institution, where the rotations took place (e.g., academic hospital belonging to the medical school, other hospitals not belonging to the medical school, public primary care clinics, etc.), and whether the school used any standardized instrument to evaluate the clerkship rotations according to the point-of-view of the medical students. If the answer to that last item was positive, we asked whether the students’ evaluations according to that instrument were anonymous and requested a copy of the assessment instrument.

We also obtained some descriptive data regarding each of the participating medical schools from the electronic registry of the Brazilian Ministry of Education. Those data included the location of the medical school (city and state), the number of years of functioning, the number of positions offered each year for new medical students, and the administrative nature of the institution (public or private).

Two independent researchers using a standardized electronic sheet extracted information concerning the assessment instruments that medical schools used to evaluate the clerkship rotations according to the perspective of students. A third researcher arbitrated any disagreements regarding data extraction.

We classified each item of the assessment instruments according to the dimension that it evaluated into one of 4 categories: a) organizational/administrative, b) technical, c) ethical, and d) nonspecific. We defined the organizational/administrative dimension as that associated with elements such as general orderliness of the rotation, neatness, punctuality of staff and activities. The technical dimension was outlined as involving aspects such as specific knowledge of diseases and the performance of diagnostic or therapeutic procedures. The ethical dimension was conceptualized as associated with moral and behavioral aspects of interpersonal relationships such as trust, confidentiality, respect, beneficence, justice, non-maleficence and autonomy. We used the nonspecific category for quite general questions that could be associated with more than one of the three previous dimensions (e.g., “How would you rate this rotation in general?”).

We also searched each instrument for items concerning the following issues:

1. Was there at least one question within the instrument that was directly related to the ethical dimension of the experiences and/or the learning that occurred during the rotation?
2. Was there at least one question concerning the performance of the preceptors of that rotation related to the ethical dimension?
3. Was there at least one question regarding the performance of multidisciplinary healthcare teams related to the ethical dimension?
4. Was there at least one question within the instrument assessing ethical implications of the physical environment where the clerkship rotations took place? For instance, if the physical environment allowed patients confidentiality and privacy when discussing sensitive issues or exposing their bodies.
5. Was there at least one question related to the occurrence of positive or negative experiences during the rotation?
6. Was there at least one question within the instrument asking students specifically about having witnessed or experienced mistreatment in the relationships involving preceptors, the multidisciplinary healthcare team, patients and/or other students?

If any of the answers to items 2, 3 or 6 above was positive, we further examined if those questions were more general or involved specific members of the team (e.g., physicians and nurses), patients and/or students.

For the purposes of data collection we defined preceptors as any healthcare professional responsible for the supervision of students during the rotations. We also defined “mistreatment” as any behavior associated with disrespect, humiliation, threats or prejudice of any kind.

We used simple measures of frequency data with 95% confidence intervals [22]. We compared means using Student’s t test and proportions with the Chi-square statistic. We also performed an exploratory multivariable logistic regression of the relationship between the presence of at least one item within the instruments concerning specifically some aspect of the ethical dimension and the following variables: years of functioning, number of vacancies for admission of new students per year and the administrative nature of each medical school (public or private).

Missing data were reported as such and were not included in the relative frequencies reported in our results.

We used the R software 3.3.2 (Vienna, 2016) [23] for all statistical analyses. We adopted 0.05 as the value of α for statistical significance.

The local Ethics Review Board of Botucatu Medical School approved the study protocol and a representative from each participating medical school provided written informed consent. We followed the STROBE guidelines [24] for presentation of reports of cross-sectional studies and a completed checklist is available as a supplementary file.

### Data

We provide the following online Supporting Information files: a de-identified study dataset (S1 File), a data dictionary (S2 File) and the research questionnaire (S3 File). Because of our confidentiality commitment with participating medical schools, we removed from the dataset any fields that could be used directly or indirectly to identify any school.

## Results

**Fig 1:**
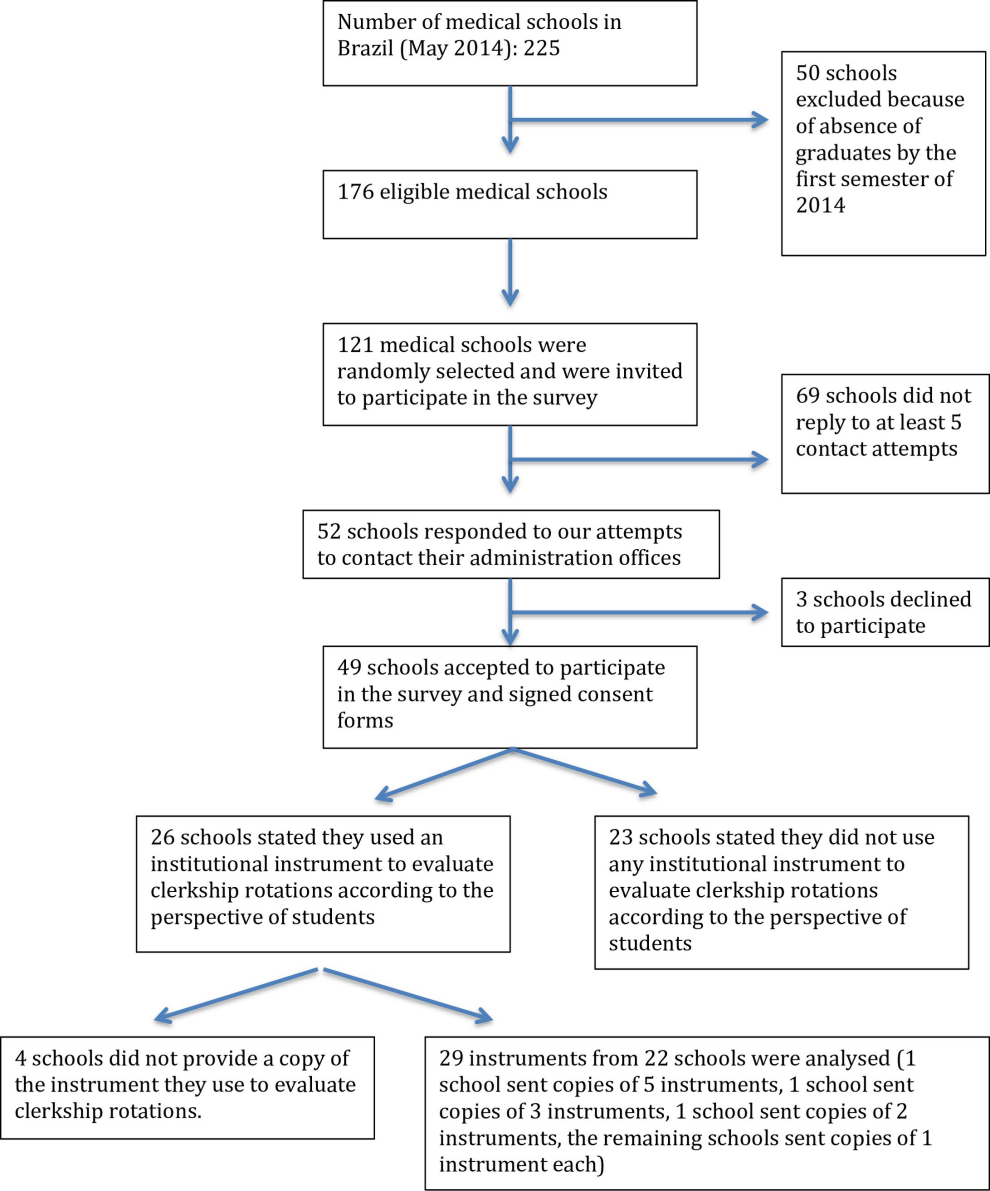
Flow diagram of the inclusion of medical schools in the study.

We provide a summary of the process of selection and invitation of medical schools to participate in the survey in Fig 1. Forty-nine (40.5%) out of 121 schools accepted our invitation to participate in the study. Nonparticipating institutions had lower mean time of functioning when compared with participating institutions (Mean Difference (MD): 35.8 years, 95%CI: 25.5–46.1, P<0.001). There were no statistically significant differences when comparing nonparticipating with participating schools concerning the mean number of vacancies for new first-year students (MD: 15.0, 95% CI: -3.0 to 33.0, P = 0.10) and the proportion of public to private schools (0.71 vs. 0.96, respectively, P = 0.54).

Of the 49 participating institutions 25 were private and 24 were public schools. The median time of functioning for all schools was 44.2 years (interquartile range (IQR): 6.7 to 51.1). For 46 schools the clerkship period lasted for 2 years, for 2 schools it lasted for 1.5 year and for 1 school it lasted for 2.5 years. All but 2 schools had at least one clerkship rotation in institutions that did not belong directly to the medical school or to its university. Only 16 schools had a university hospital of their own and 39 schools used other hospitals outside of its direct administration as a training field for at least one rotation. Forty-five schools used public primary care clinics as a training field for their students.

Twenty-six (53.1%, 95%CI: 39.4–66.3%) schools reported using at least one institutional instrument to evaluate the quality of clerkship rotations according to the perspective of students. Twenty-four (92.3%, 95%CI: 75.9–97.9%) of those 26 schools stated that assessment instruments allowed student anonymity. However, we could confirm that the assessment instruments were clearly anonymous (e.g., there was no field for student identification) for just 12 (54.5%, 95%CI: 34.7–73.1%) of the 22 schools that had sent us copies of those documents. For 4 (18.2%, 95%CI: 7.3–38.5%) other medical schools there was some written information within their instruments stating that student identification was optional.

Table 1 describes the composition of the assessment instruments according to the 4 dimensions we adopted for our analyses. Table 2 shows examples of questions regarding those 4 dimensions within the instruments analyzed.

**Table 1:** Mean numbers of items by assessment instrument and overall number of items among the 29 assessment instruments analysed according to the dimension addressed by each item.

Dimension	Mean (SD)	N (%)
Ethical	1.6 (1.8)	36 (7.7%)
Organizational	8.5 (12.1)	186 (40%)
Technical	7.1 (8.9)	156 (33.5%)
Nonspecific	4.0 (3.6)	87 (18.8%)
Total	21.1 (23.4)	465 (100%)

**Table 2:** Examples of items addressing ethical, technical, organizational/administrative and nonspecific dimensions within the instruments analysed.

**Ethical** • Did your preceptor foster an ethical and professional posture? • Was your preceptor able to receive and offer criticism with serenity and respectfully? • Is the relationship between your preceptor and patients based upon respect and understanding? • Have you witnessed any situations where patients were mistreated? • Were there any situations where the medical code of ethics was not honored? **Organizational/administrative** • Did you receive sufficient information about the program, your duties, the learning objectives and learning resources in the first day of the rotation? • The criteria for the evaluation of the students’ performance were clear? • Was the time assigned to different activities (e.g., ambulatory clinics and operating room) sufficient during the rotation? Technical • How would you rate the proficiency of your preceptor concerning the subjects that were discussed? • The rounds added to your medical knowledge? • Were the theoretical and practical aspects of medicine integrated appropriately during the rotation? **Nonspecific** • How would you rate this rotation in general? • What were the strengths and weaknesses of this rotation? • In a 1 to 10 scale, how would you rate the overall performance of your preceptor during the rotation?

Only 13 (26.5%, 95%CI: 16.2–40.3%) of 49 schools had an instrument with at least one item addressing issues related to the ethical dimension. The most common items associated with that dimension involved the relationship between preceptors and students (13 schools, 26.5%, 95%CI: 16.2–40.3%), preceptors and patients/families (7 schools, 14.3%, 95%CI: 7.1%-26.7%), preceptors and the multidisciplinary healthcare team (3 schools). The instruments of only 2 (4.1%, 95%CI: 1.1–13.7%) schools included 1 question regarding specifically the experience of negative ethical events during a rotation and just 1 (2.0%, 95%CI: 0.4–10.7%) school asked for positive ethical experiences. Likewise, merely 1 medical school specifically asked if students had witnessed any situation of patient mistreatment during a rotation, and no similar questions were made concerning mistreatment against the students themselves or towards members of the healthcare team. Additionally, no school (0%, 95%CI: 0–7.3%) asked students about aspects of the physical environment associated with the ethical dimension, such as if it allowed for privacy.

The exploratory logistic regression did not disclose any significant relationship between the presence of at least one item within the questionnaires concerning specifically some aspect of the ethical dimension and the following variables: years of functioning (OR: 1.00, 95%CI: 0.98–1.02), number of vacancies for admission of new students per year (OR: 1.00, 95%CI: 0.99–1.02) and the administrative nature of each medical school being public instead of private (OR: 0.81, 95%CI: 0.19–3.49).

## Discussion

To our knowledge this is the first national survey of medical schools in any country focusing specifically on the institutional instruments used to assess the quality of clerkship rotations according to the perspective of medical students. The first relevant finding of our research was that almost half of the participating medical schools did not have any institutional instrument designed to assess the quality of the clerkship rotations according to the perspective of students. That finding is important because it discloses a major missed opportunity to unveil aspects of institutions and of their functioning that could benefit from quality improvement initiatives concerning both medical education and the care of patients. It is especially relevant in the present context where most schools have several rotations taking place outside their institutions. It also represents a wasted chance to communicate students and staff about the most important values and quality models that schools want to nurture within their domains in a more concrete manner than through institutional discourses.

Another important result lies in the absence of several ethics-related items within the instruments that we analyzed. For instance, the finding that only 13 of 29 instruments had at least one question encompassing the ethical dimension suggests that there is often a divide between the usual emphasis that medical schools give to that dimension in their official discourse and the actual importance it receives within the hidden curricula of institutions. That interpretation is consistent with the results from Stern and colleagues [25] who showed significant differences between the “recommended curriculum” and the one actually taught in inpatient general medical wards. Our results allow us to hypothesize that the dearth of assessment instruments emphasizing the ethical behaviors recommended within formal curricula might be a contributing factor for the phenomenon they described.

There is a plethora of evidence demonstrating that anti-ethical behaviors such as harassment and other forms of mistreatment are common in healthcare institutions around the world [9,15,16,26–29]. Despite that fact, only 2 instruments asked students directly about negative ethical experiences during the rotations and only one school asked if students had witnessed any occurrence of disrespect towards patients. No school asked students whether they had either experienced or witnessed mistreatment toward themselves, other students or staff members. When schools fail to ask students specifically about those issues within their institutional assessment framework, they are turning a blind eye to those unfortunate situations, which certainly facilitates their perpetuation.

A possible explanation for the absence of ethics-related questions is that many leaders and administrators of medical institutions were not trained about how to confront such problems and end up choosing not to face those issues [30].

Our findings are in marked contrast with the American Academy of Medical Colleges Graduation Questionnaire, which has an entire section devoted to “Behaviors experienced during medical school” where students are asked straightforwardly for mistreatment experiences ranging from public humiliation to sexual harassment [31,32].

It was reassuring to notice that most (24 out of 26) medical schools understood their assessment instruments allowed for student anonymity. This is important because mechanisms that allow for anonymity may decrease fear of retaliation, which is a significant barrier for the reporting of mistreatment and disruptive behaviors by faculty and staff [33–36]. However, we were not able to analyze how effectively anonymity was ensured by internal procedures within each institution, especially in the 10 cases of medical schools where there was a field for student identification within the assessment instruments.

The absence of any clear relationship between the presence of at least one item addressing the ethical dimension within the instruments that we evaluated and the number of years of functioning, the number of vacancies for admission of new students per year and the administrative nature of each medical school was not surprising. It is probably a consequence not only of the relatively limited number of participating schools but more likely of the fact that the characteristics of the assessment instruments from individual institutions depend on much more complex variables than we were able to measure.

Although our results are restricted to Brazilian medical schools, reports from other areas of the world have also identified several problems concerning numerous aspects of medical education, ranging from the use of outdated sources of information and lack of appropriate supervision of medical students to fraud [37–41]. Furthermore, the rapid growth in the numbers of new medical schools in the world has raised concerns that many of them might have started functioning on inappropriate grounds [42]. Hence, we hypothesize that the dearth of assessment instruments and of ethics-related items within instruments that we found in this study may also be common in other countries.

This study has several potential limitations. The participation ratio of medical schools was much below the intended number and due to logistical reasons and limitations regarding funding we were not able to expand data collection to schools outside our initial randomization list. Therefore, our results may not be generalizable to the remaining medical schools in the country. However, it is probable that nonparticipating schools were more likely not to have any institutional instrument to assess clerkship rotations, since that situation could have contributed to their nonparticipation and because nonparticipating schools had significantly less years of functioning than participating schools. Hence, our results might represent a conservative and overoptimistic picture. We adopted a very broad conceptualization of the ethical dimension during the analysis of the assessment instruments. For instance, if instruments had any question about the quality of the interpersonal relationships between preceptors and students, they were classified as having at least one item addressing the ethical dimension. In addition, we were not able to assess how the ethical dimension is assessed in other courses of participating medical schools or the existence of other initiatives that were designed to allow the reporting of harassment, mistreatment and other types of unethical behavior (e.g. ethics hotlines). Finally, we were not able to examine how medical schools deal with the results from the assessments they received from students concerning clerkship rotations or other strategies in place to promote an ethical cultural learning environment.

In summary, our results show there are several missed opportunities in the way medical schools assess the quality of clerkship rotations regarding ethical domains. Closing the gap between usual institutional discourses regarding ethics and how those dimensions are assessed within clerkship rotations might represent an important step towards the improvement of medical education and healthcare systems.

## Supporting information

S1 File De-identified dataset. (XLSX)

S2 File Data dictionary. (DOCX)

S3 File Research questionnaire. (DOCX)

## References

[1] Dyrbye LN, Massie FS Jr, Eacker A, Harper W, Power D, Durning SJ, et al Relationship between burnout and professional conduct and attitudes among US medical students. JAMA. 2010;304: 1173–1180. 10.1001/jama.2010.1318 20841530

[2] Gaiser RR. The teaching of professionalism during residency: why it is failing and a suggestion to improve its success. Anesth Analg. 2009;108: 948–954. 10.1213/ane.0b013e3181935ac1 19224808

[3] Leape LL, Shore MF, Dienstag JL, Mayer RJ, Edgman-Levitan S, Meyer GS, et al Perspective: a culture of respect, part 1: the nature and causes of disrespectful behavior by physicians. Acad Med. 2012;87: 845–852. 10.1097/ACM.0b013e318258338d 22622217

[4] Neumann M, Edelhäuser F, Tauschel D, Fischer MR, Wirtz M, Woopen C, et al Empathy decline and its reasons: a systematic review of studies with medical students and residents. Acad Med. 2011;86: 996–1009. 10.1097/ACM.0b013e318221e615 21670661

[5] Hojat M, Vergare MJ, Maxwell K, Brainard G, Herrine SK, Isenberg GA, et al The devil is in the third year: a longitudinal study of erosion of empathy in medical school. Acad Med. 2009;84: 1182–1191. 10.1097/ACM.0b013e3181b17e55 19707055

[6] Feudtner C, Christakis DA, Christakis NA. Do clinical clerks suffer ethical erosion? Students’ perceptions of their ethical environment and personal development. Acad Med. 1994;69: 670–679. 8054117 10.1097/00001888-199408000-00017

[7] Leape LL, Shore MF, Dienstag JL, Mayer RJ, Edgman-Levitan S, Meyer GS, et al Perspective: a culture of respect, part 2: creating a culture of respect. Acad Med. 2012;87: 853–858. 10.1097/ACM.0b013e3182583536 22622219

[8] Makary MA, Daniel M. Medical error—the third leading cause of death in the US. BMJ. 2016;353: i2139 10.1136/bmj.i2139 27143499

[9] Hicks LK, Lin Y, Robertson DW, Robinson DL, Woodrow SI. Understanding the clinical dilemmas that shape medical students’ ethical development: questionnaire survey and focus group study. BMJ. 2001;322: 709–710. 10.1136/bmj.322.7288.709 11264209 PMC30097

[10] Roff S, Preece P. Helping medical students to find their moral compasses: ethics teaching for second and third year undergraduates. J Med Ethics. 2004;30: 487–489. 10.1136/jme.2003.003483 15467084 PMC1733931

[11] D’Eon M, Lear N, Turner M, Jones C. Perils of the hidden curriculum revisited*. Med Teach. 2007;29: 295–296. 10.1080/01421590701291485 17786739

[12] Hafferty FW. Beyond curriculum reform: confronting medicine’s hidden curriculum. Acad Med. 1998;73: 403–407. 10.1097/00001888-199804000-00013 9580717

[13] Silver HK, Glicken AD. Medical student abuse. Incidence, severity, and significance. JAMA. 1990;263: 527–532. 10.1001/jama.1990.03440040066030 2294324

[14] Sheehan KH, Sheehan DV, White K, Leibowitz A, Baldwin DC Jr. A pilot study of medical student “abuse”. Student perceptions of mistreatment and misconduct in medical school. JAMA. 1990;263: 533–537. 10.1001/jama.1990.03440040072031 2294325

[15] Fnais N, Soobiah C, Chen MH, Lillie E, Perrier L, Tashkhandi M, et al Harassment and discrimination in medical training: a systematic review and meta-analysis. Acad Med. 2014;89: 817–827. 10.1097/ACM.0000000000000200 24667512

[16] Mavis B, Sousa A, Lipscomb W, Rappley MD. Learning about medical student mistreatment from responses to the medical school graduation questionnaire. Acad Med. 2014;89: 705–711. 10.1097/ACM.0000000000000199 24667505

[17] O’Sullivan H, van Mook W, Fewtrell R, Wass V. Integrating professionalism into the curriculum: AMEE Guide No. 61. Med Teach. 2012;34: 64–77. 10.3109/0142159X.2012.655610 22288994

[18] Wilkes M, Bligh J. Evaluating educational interventions. BMJ. 1999;318: 1269–1272. 10.1136/bmj.318.7193.1269 10231263 PMC1115653

[19] Morrison J. ABC of learning and teaching in medicine: Evaluation. BMJ. 2003;326: 385–387. 10.1136/bmj.326.7385.385 12586676 PMC1125244

[20] Wormald BW, Schoeman S, Somasunderam A, Penn M. Assessment drives learning: an unavoidable truth? Anat Sci Educ. 2009;2: 199–204. 10.1002/ase.102 19743508

[21] Fleiss JL, Levin BA, Paik MC. Statistical methods for rates and proportions Hoboken, N.J.: J. Wiley; 2003.

[22] Altman D, Machin D, Bryant T, Gardner M, editors. Statistics with Confidence: Confidence Intervals and Statistical Guidelines 2 edition London: BMJ Books; 2000.

[23] R core team. R: A language and environment for statistical computing [Internet]. Vienna, Austria: R Foundation for Statistical Computing; 2017. Available: https://www.R-project.org/

[24] von Elm E, Altman DG, Egger M, Pocock SJ, Gøtzsche PC, Vandenbroucke JP, et al Strengthening the Reporting of Observational Studies in Epidemiology (STROBE) statement: guidelines for reporting observational studies. BMJ. 2007;335: 806–808. 10.1136/bmj.39335.541782.AD 17947786 PMC2034723

[25] Stern DT. Practicing what we preach? An analysis of the curriculum of values in medical education. Am J Med. 1998;104: 569–575. 10.1016/S0002-9343(98)00109-0 9674721

[26] Brainard AH, Brislen HC. Viewpoint: Learning Professionalism: A View from the Trenches: Acad Med. 2007;82: 1010–1014. 10.1097/01.ACM.0000285343.95826.94 17971682

[27] Nagata-Kobayashi S, Maeno T, Yoshizu M, Shimbo T. Universal problems during residency: abuse and harassment. Med Educ. 2009;43: 628–636. 10.1111/j.1365-2923.2009.03388.x 19573185

[28] Satterwhite RC, Satterwhite WM, Enarson C. An ethical paradox: the effect of unethical conduct on medical students’ values. J Med Ethics. 2000;26: 462–465. 10.1136/jme.26.6.462 11270946 PMC1733313

[29] Mullan CP, Shapiro J, McMahon GT. Interns’ experiences of disruptive behavior in an academic medical center. J Grad Med Educ. 2013;5: 25–30. 10.4300/JGME-D-12-00025.1 24404222 PMC3613313

[30] Vidal EI de O, Silva V dos S, Santos MF dos, Jacinto AF, Boas PJFV, Fukushima FB. Why Medical Schools Are Tolerant of Unethical Behavior. Ann Fam Med. 2015;13: 176–180. 10.1370/afm.1763 25755040 PMC4369591

[31] American Academy of Medical Colleges. Medical School Graduation Questionnaire: 2015 All Schools Summary Report [Internet]. 2015 [cited 21 Feb 2017]. Available: https://www.aamc.org/download/440552/data/2015gqallschoolssummaryreport.pdf

[32] Dickstein LJ, Savoia M, Culbert AJ, Dobbins D, Hall. Appropriate treatment in medicine (ATM): a compendium on medical student mistreatment: a project of the AAMC Group on Student Affairs [Internet] Washington, D.C.: AAMC; 2000 Available: https://www.aamc.org/download/113886/data/atm-spring2000.pdf.pdf

[33] Shapiro J, Whittemore A, Tsen LC. Instituting a culture of professionalism: the establishment of a center for professionalism and peer support. Jt Comm J Qual Patient Saf. 2014;40: 168–177. 10.1016/S1553-7250(14)40022-9 24864525

[34] Dupree E, Anderson R, McEvoy MD, Brodman M. Professionalism: a necessary ingredient in a culture of safety. Jt Comm J Qual Patient Saf. 2011;37: 447–455. 10.1016/S1553-7250(11)37057-2 22013818

[35] Hickson GB, Pichert JW, Webb LE, Gabbe SG. A complementary approach to promoting professionalism: identifying, measuring, and addressing unprofessional behaviors. Acad Med. 2007;82: 1040–1048. 10.1097/ACM.0b013e31815761ee 17971689

[36] Speck RM, Foster JJ, Mulhern VA, Burke SV, Sullivan PG, Fleisher LA. Development of a professionalism committee approach to address unprofessional medical staff behavior at an academic medical center. Jt Comm J Qual Patient Saf. 2014;40: 161–167. 10.1016/S1553-7250(14)40021-7 24864524

[37] Jsrgin SV. Some Aspects of Medical Education in Russia. Am J Med Stud. 2013;1: 4–7. 10.12691/ajms-1-2-1

[38] Aggarwal S, Sharma V. The problems of medical education in a developing economy: The case of India. Ann Trop Med Public Health. 2012;5: 627–629. 10.4103/1755-6783.109346

[39] MacAskill A, Stecklow S, Miglani S. Special Report: Why India’s medical schools are plagued with fraud. Reuters. 17 Jun 2015. Available: http://www.reuters.com/article/us-india-medicine-education-specialrepor-idUSKBN0OW1NM20150617. Accessed 25 Feb 2017.

[40] Celletti F, Buch E, Samb B. Medical education in developing countries In: Walsh K, editor. Oxford Textbook of Medical Education. Oxford University Press; 2013 pp. 671–682. 10.1093/med/9780199652679.003.0057

[41] Deswal BS, Singhal VK. Problems of medical education in India. Int J Community Med Public Health. 2016;3: 1905–1909. 10.18203/2394-6040.ijcmph20162063

[42] World Federation for Medical Education. Basic Medical Education: WFME Global Standards for Quality Improvement: The 2015 Revision [Internet]. Ferney-Voltaire: WFME Office; 2015. Available: http://wfme.org/standards/bme/78-new-version-2012-quality-improvement-in-basic-medical-education-english/file

