# Supplementary material for: Missed opportunities in the way medical schools evaluate the ethical domain in clerkship rotations
Source: PLoS One. 2019 May 29;14(5):e0217717. doi: 10.1371/journal.pone.0217717 (PMC6541282; doi:10.1371/journal.pone.0217717)
Supplement: S2 File — (DOCX) [file pone.0217717.s002.docx]

**DATA DICTIONARY**

A- MEDICAL_SCHOOL: Idendification code for each participating school.

B- PUBLIC_PRIVATE: Public x Private medical schools

C- HAS_AN_INSTRUMENT: 1 if the medical school has an assessment instrument and 0 if doesn’t have.

D- ANONYMOUS: 1 if the instrument does not require student identification and 0 if otherwise.

E- GENERAL_ETHICS: 1 if the instrument addresses any issues connected to the ethical dimensions and 0 if otherwise.

F- STUDENT_SELF_EVALUATION: 1 if the form asks students to self-evaluate their ethical learning and / or behavior during the rotation; 0 if otherwise.

G- PRECEPTOR_IN_GENERAL: 1 if the form asks students to evaluate preceptors in relation to ethical behavior in general.

H- PRECEPTOR_PATIENT: 1 if the form asks students to evaluate the behavior of preceptors towards patients in ethical terms and 0 if otherwise.

I- PRECEPTOR_STUDENT: 1 if the form asks students to evaluate the behavior of preceptors towards students in ethical terms and 0 if otherwise.

J- PRECEPTOR_TEAM: 1 if the form asks students to evaluate the behavior of preceptors towards the healthcare team in ethical terms, and 0 if otherwise.

K- TEAM_IN_GENERAL: 1 if the form asks students to evaluate the behavior of the team in general regarding the ethical stance, and 0 if otherwise.

L- TEAM_PATIENT: 1 if the form asks students to evaluate the behavior of the team towards patients in ethical terms, and 0 if otherwise.

M- TEAM_STUDENT: 1 if the form asks students to evaluate the behavior of the team towards students in ethical terms, and 0 if otherwise.

N- PHYSICAL_ENVIRONMENT: 1 if the form asks students to evaluate any aspect of the physical environment of the institution in ethical terms, and 0 if otherwise.

O- GENERAL_ EVALUATION_CLERKSHIP): 1 if the form asks students to evaluate the clerkship rotation in general without other specifications, and 0 if otherwise.

P- SELF_EVALUATION_TECHNICAL_LEARNING: 1 if the form asks students to self-evaluate his / her degree of technical learning during the clerkship rotation, and 0 if otherwise.

Q- SELF-EVALUATION_INTERPERSONAL: 1 if the form asks students to self-evaluate their behavior regarding the relationships with other people (i.e. patients, preceptor, other students and members of the healthcare team) during the clerkship and 0 if otherwise.

R- THEORY_PRACTICAL_CORRELATION: 1 if the form asks students to evaluate if the clerkship allowed them to correlate theory with practical applications, and 0 if otherwise.

S- ORGANIZATIONAL: 1 if the form asks students to evaluate organizational aspects of the clerkship rotation and 0 if otherwise.

T- QUALITY_OF_EVALUATION: 1 if the form asks students to evaluate the quality of the evaluation process to which they were submitted during the clerkship rotation (e.g. practical tests, theoretical tests, formal feedback from the preceptor) and 0 if otherwise.

U- PRECEPTOR_TECHNICAL_COMPETENCE: 1 if the form asks the student to evaluate preceptors regarding technical competence, and 0 if otherwise.

V- CLERKSHIP_TECNICAL_LEARNING: 1 if the form asks students to evaluate the adequacy of the technical learning to the practical activities developed during the clerkship rotation, and 0 if otherwise.

W- STRENGTHS: 1 if the form asks students to point out the strengths of the clerkship rotation, and 0 if otherwise.

X- WEAKNESSES: 1 if the form asks students to point out the weaknesses (negative, critics) of the clerkship rotation, and 0 if otherwise.

Y- OPEN_QUESTIONNAIRE: 1 if the questionnaire is composed exclusively by open questions and 0 if otherwise.

Z- CLOSED_QUESTIONNAIRE: 1 if the questionnaire is composed exclusively by closed questions and 0 if otherwise.

AA- MIXED_QUESTIONNAIRE: 1 if the questionnaire is composed by closed and open questions and 0 if otherwise.

AB- PRECEPTOR_NON-SPECIFIC: 1 if there is any field within the form where students are asked to provide a general and non-specific evaluation of the preceptor that does not specifically involve issues related to ethics or to technical competence; 0 if otherwise.

AC- SUGGESTIONS: 1 if there is any field asking students to provide free comments, observations and suggestions regarding the clerkship rotation; 0 if otherwise.

AD- NEGATIVE_ETHICAL_EXPERIENCES: 1 if there is any field that specifically inquires students about the occurrence of ethically negative experiences during the clerkship rotation; 0 if otherwise.

AE- POSITIVE_ETHICAL_EXPERIENCES: 1 if there is any field that specifically inquires students about the occurrence of ethically positive experiences during the clerkship rotation; 0 if otherwise.

AF- MISTREATMENT_STUDENTS: 1 if there is any field that specifically inquires students about any experiences of mistreatment against students during the clerkship rotation; 0 if otherwise.

AG- MISTREATMENT_PATIENTS: 1 if there is any field that specifically inquires students about experiences of mistreatment against patients during the clerkship rotation; 0 if otherwise.

AH- MISTREATMENT_TEAM: 1 if there is any field that specifically inquires students about experiences of mistreatment against members of the healthcare team during the clerkship rotation; 0 if otherwise.

AI- GENERAL_STRUCTURE: 1 if the form asks students to evaluate any aspect of the physical structure not related to the ethical dimension; 0 if otherwise.

AJ- TEAM_TECHNICAL_COMPETENCE: 1 if the form asks students to evaluate the healthcare team present in the clerkship rotation as a whole or in the form of any professional other than their preceptors in relation to technical competence; 0 if otherwise.

AK- N_ORGANIZATIONAL: Number of items referring to the organizational-administrative dimension within the instrument.

AL- N_TECHNICAL: Number of items referring to the technical dimension within the instrument.

AM- N_ETHICAL: Number of items referring to the ethical dimension within the instrument.

AN- N_NONSPECIFIC: Number of non-specific items within the instrument.

AO- N_TOTAL: Total number of items within the instrument.
