# Supplementary material for: Missed opportunities in the way medical schools evaluate the ethical domain in clerkship rotations
Source: PLoS One. 2019 May 29;14(5):e0217717. doi: 10.1371/journal.pone.0217717 (PMC6541282; doi:10.1371/journal.pone.0217717)
Supplement: S3 File — (DOCX) [file pone.0217717.s003.docx]

**Research form to be completed by the person indicated by the Dean or Vice-dean of the medical school:**

1) Name and position of person responsible for providing information:

1a) Name: _________________________________________________________________________________

1b) Position or function: ____________________________________________________________________

2) Name of medical school: ____________________________________________________________

3) Medical school location: City ___________________________________________, State: ___

4) What is the total length of the clerkship period in this institution?

() 1 year () 1,5 year () 2 years () 2.5 years () Other: _________________

5) Please indicate all the alternatives currently used by the students in your institution with regards to clerkship rotations in your institution:

(a) University Hospitals belonging to the medical school

(b) Public hospitals not belonging to the medical school

(c) Private hospitals not belonging to the medical school

(d) Primary Health Care Units

(e) Public outpatient specialty clinics

(f) Private outpatient specialty clinics

(g) Others. Please, specify: _____________________________________________________________

(h) None of the students of this institution has already reached the moment of starting clerkship, since this school has been active for less than five years.

6) Does this medical school use any institutional instruments that are completed by the students so that they may evaluate their clerkship rotations?

() Yes () No () I don’t know

7) In case of a positive answer to the previous question, is this instrument completed anonymously by the students?

() Yes () No () I do not know

8) In the event of an affirmative answer to item 6 and as previously authorized by the Dean of Vice-dean of this institution, we request that a copy of this instrument be sent to the e-mail: [pesquisa.avaliacao.internato@gmail.com](mailto:pesquisa.avaliacao.internato@gmail.com) or to FAX: 55-14-3882-2238 to Prof. Edison Iglesias de Oliveira Vidal

**Formulário da pesquisa a ser preenchido pela pessoa indicada pelo diretor ou vice-diretor da escola médica:**

1) Nome e cargo do responsável pelo fornecimento das informações:

1a) Nome:_________________________________________________________________________________

1b) Cargo ou função: ____________________________________________________________________

2) Nome da escola médica: ____________________________________________________________

3) Local da escola médica: Cidade ___________________________________________, UF:___

4) Qual a duração total do período de internato nesta instituição?

( ) 1 ano ( ) 1,5 ano ( ) 2 anos ( ) 2,5 anos ( ) outro: _________________

5) Por favor, em relação ao local de realização dos estágios de internato em sua instituição, assinale todas as alternativas atualmente utilizadas pelos estudantes em sua instituição:

(a) Hospitais Universitários pertencentes à escola médica

(b) Hospitais públicos não-pertencentes à escola médica

(c) Hospitais privados não-pertencentes à escola médica

(d) Rede de Atenção Básica à saúde

(e) Ambulatórios públicos de especialidades

(f) Ambulatórios privados de especialidades

(g) outros. Especificar: _____________________________________________________________

(h) Nenhuma das turmas de alunos desta instituição já alcançou o momento de iniciar os estágios de internato, uma vez que se trata de escola que entrou em atividade há menos de cinco anos.

6) Esta escola médica utiliza algum instrumento institucional preenchido pelos alunos para que estes possam avaliar seus estágios de internato?

( ) Sim ( ) Não ( )Desconheço

7) Em caso de resposta afirmativa para a pergunta anterior, este instrumento é preenchido de forma anônima pelos alunos?

( ) Sim ( ) Não ( ) Desconheço

8) Ainda em caso de resposta afirmativa para o item 6 e conforme previamente autorizado pelo diretor ou vice-diretor desta instituição, solicitamos que uma cópia deste instrumento seja enviada para o correio eletrônico: [pesquisa.avaliacao.internato@gmail.com](mailto:pesquisa.avaliacao.internato@gmail.com) ou para o FAX: 14- 3882-2238 aos cuidados do Prof. Edison Iglesias de Oliveira Vidal
